# Myalgic Encephalomyelitis/Chronic Fatigue Syndrome (ME/CFS) and Comorbidities: Linked by Vascular Pathomechanisms and Vasoactive Mediators?

**DOI:** 10.3390/medicina59050978

**Published:** 2023-05-18

**Authors:** Klaus J. Wirth, Matthias Löhn

**Affiliations:** Institute of General Pharmacology and Toxicology, University Hospital Frankfurt am Main, Goethe-University, Theodor-Stern Kai 7, D-60590 Frankfurt am Main, Germany; wirth@em.uni-frankfurt.de

**Keywords:** ME/CFS, long COVID, MCA, endometriosis, dysmenorrhea, orthostatic intolerance, small fiber neuropathy, cerebral blood flow, brain fog, β2-adrenergic receptors

## Abstract

Myalgic Encephalomyelitis/Chronic Fatigue Syndrome (ME/CFS) is often associated with various other syndromes or conditions including mast cell activation (MCA), dysmenorrhea and endometriosis, postural tachycardia (POTS) and small fiber neuropathy (SFN). The causes of these syndromes and the reason for their frequent association are not yet fully understood. We previously published a comprehensive hypothesis of the ME/CFS pathophysiology that explains the majority of symptoms, findings and chronicity of the disease. We wondered whether some of the identified key pathomechanisms in ME/CFS are also operative in MCA, endometriosis and dysmenorrhea, POTS, decreased cerebral blood flow and SFN, and possibly may provide clues on their causes and frequent co-occurrence. Our analysis indeed provides strong arguments in favor of this assumption, and we conclude that the main pathomechanisms responsible for this association are excessive generation and spillover into the systemic circulation of inflammatory and vasoactive tissue mediators, dysfunctional β2AdR, and the mutual triggering of symptomatology and disease initiation. Overall, vascular dysfunction appears to be a strong common denominator in these linkages.

## 1. Introduction

Chronic Fatigue Syndrome or Myalgic Encephalomyelitis (ME/CFS) is a frequent, debilitating and enigmatic disease. ME/CFS presents with a confusing myriad of symptoms ranging from neurological symptoms, fatigue, exertional intolerance with post-exertional malaise (PEM), chronic muscle pain, skeletal muscle and cardiovascular findings to complaints arising from many organs [1]. ME/CFS is often triggered by various viral infections, among them Epstein–Barr virus (EBV), human herpesvirus 6 (HHV-6), enteroviruses, influenza, dengue fever and the corona virus SARS-CoV-2 [2,3]. Its prevalence is supposed to strongly rise due to COVID-19.

ME/CFS is associated with various syndromes, diseases or conditions, including mast cell activation (MCA) [4,5], endometriosis (EM) [6,7,8,9,10], postural orthostatic tachycardia syndrome (POTS) [11,12], orthostatic intolerance (OI) [13], and small fiber neuropathy (SFN) [12,14]. Even when diagnosed independently of each other, ME/CFS, endometriosis and MCA share many symptoms, such as fatigue, brain fog, cognitive impairment, pain and edema, just to name a few. The causes for these associations are unclear. We have published a unifying and comprehensive hypothesis of the pathophysiology of ME/CFS [5]. This hypothesis allows us to explain the majority of symptoms, findings and chronicity of ME/CFS, but in addition also provides possible hints for the frequently observed association with these other syndromes. Here, we try to further analyze the causes for these associations, starting from the assumption that similar pathomechanisms as identified in our disease hypothesis for ME/CFS are also effective in the other syndromes.

Mast cell activation syndrome and endometriosis are commonly recognized as independent syndromes that can be associated with ME/CFS. Postural tachycardia (POTS), orthostatic intolerance (OI) and SFN (SFN) seem to be more closely related to ME/CFS and are rather considered as risk factors, premorbid conditions or part of the ME/CFS symptomatology, although these syndromes also may exist independently of ME/CFS. Since the relationships of these syndromes to ME/CFS seem to be different, in the following, we discuss possible similarities and differences in pathomechanisms for both groups of syndromes separately.

## 2. Common Pathomechanisms in ME/CFS, MCA and Dysmenorrhea

### 2.1. Excessive Generation of Tissue Inflammatory Mediators and Spillover into the Systemic Circulation Causes Symptoms

In our original hypothesis, we consider ME/CFS as a disease in which the spillover of tissue mediators from the affected skeletal muscles into the systemic circulation plays a key role [5]. Inflammatory and vasoactive mediators such as bradykinin, prostaglandins, prostacyclin, ATP and adenosine are released from working muscles already in the physiological process of functional sympatholysis and even more so when the muscles are in a state of low energy such as in ischemia or mitochondrial dysfunction [5]. These tissue mediators are physiologically meant to act only locally in the muscle due to a very short half-life to efficiently raise muscle perfusion. In ME/CFS, the metabolically driven generation of these mediators in the beginning of muscle work or in hypoxia becomes excessive due to a predominance of vasoconstrictor over vasodilator influences, resulting in hypoperfusion (which is then quickly compensated by these mediators) and a poor metabolic situation in skeletal muscle. The latter occurs as consequence of a metabolic disturbance, which is mainly due to mitochondrial dysfunction causing an energy deficit that stimulates the production of such mediators for compensation. Excessive production of these vasoconstrictor mediators during muscle work and even mental stress may cause their spillover into the systemic circulation so that they can reach any organ in the body to trigger a myriad of symptoms such as pain, hyperalgesia, spasms and edema according to their physiological effects, and also to cause a special renal situation (renal hyperexcretion) with hypovolemia but, paradoxically, low renin. The rise in renal perfusion and in tubular sodium concentration stimulated by the renal vasodilator and natriuretic effect of mediators such as bradykinin and prostacyclin annihilates the signal for renin production explaining the missing rise in renin as a response to hypovolemia and the renin paradox [5].

### 2.2. Association of MCA and ME/CFS and Common Pathomechanisms

MCA may be difficult to diagnose clinically, but the symptoms arising from mast cell degranulation such as diarrhea, hypotension, urticaria, nasal obstruction and asthma are easily understood based on the physiological actions of the released mediators. Here, we use the term “mast cell activation (MCA)” instead of “mast cell activation syndrome (MCAS)” because the latter is narrowly defined and requires the demonstration of elevated tryptase levels and organ manifestations such as urticaria. The association of MCAS and ME/CFS is infrequent [15]. Nevertheless, there is clear evidence for a role of histamine and allergic predisposition in ME/CFS and long COVID. The most convincing evidence for the involvement of histamine is the alleviating action of long-term symptoms of post-COVID-19 infection upon the anti-histamine treatment [16,17,18,19].

Upon contact to various stimuli, mast cells release many mediators, including histamine, heparin, prostaglandins, leukotrienes and different proteases (tryptase, chymase), and more than 30 cytokines and chemokines [20]. Moreover, upon inappropriate activation of mast cells by an altered activation threshold or altered expression of receptors, the spillover of released mediators into the adjacent tissue(s) and/or blood circulation may occur [20]. Most of the mediators are rapidly degraded, which makes their diagnostics complicated. The physiological function of mediators released from mast cells is the maintenance of tissue homeostasis and their effects in the tissue should be appropriate and limited to it. Excessive generation of mast cell mediators to an extent that effects occur in remote organs is pathologic, causing anaphylaxis and asthma, for example. It has been hypothesized that COVID-19 infection could lead to an exaggeration of a pre-existing but undiagnosed MCA and that MCA symptoms were increased in severity in long COVID, which may reflect an increased activation of mast cells upon SARS-CoV-2 infection [21,22].

### 2.3. Link of Endometriosis/Dysmenorrhea and ME/CFS

Endometriosis (EM) has been recognized as the presence of endometrial tissue outside the uterine cavity and a chronic inflammatory, estrogen-dependent gynecological disease. Interestingly, EM is a comorbid condition in women with ME/CFS, with more than one third of affected females also having a diagnosis of dysmenorrhea or endometriosis [6,23]. The comorbidity was associated with chronic pelvic pain, earlier menopause, hysterectomy, and more ME/CFS-related symptoms [6,7,8,9]. Multiple attempts have been made in order to identify an inflammatory cytokine profile which could serve as a diagnostic marker and would provide targets to develop appropriate therapeutics. However, the published results are inconsistent and have not identified clinically useful biomarkers to date [7,24].

In dysmenorrhea, the uterus becomes ischemic due to hypercontractility precluding perfusion for the time of the contractions because of the high intramural pressure which then leads to the ischemically stimulated release of compensatory vasoactive mediators to restore perfusion [25]. Excessive production presumably then could lead to a spillover of these mediators into the systemic circulation so that these algesic, spasmogenic vasoactive mediators (vasodilator and microvascular leakage increasing) cause symptoms such as spasms, and edema pain in almost every organ as if an intravenous infusion of a cocktail of such mediators would be performed. Similarly, mediators such as PGE2 may also be released from endometriotic foci located outside the uterine cavity, e.g., in the peritoneal cavity, ovaries, bladder or ureters [26,27,28], and to cause irregular symptoms in these tissues. These mediators, when released into the blood stream, apart from reaching other organs, can still enhance uterine contractility, particularly via PGE2, and thus cause uterine ischemia by provoking hypercontractility which then leads to the generation of ischemically stimulated vasoactive mediators, and when excessively produced may also reach the systemic circulation.

Similar mechanisms of vasoactive tissue mediator generation have also been described in other organs, such as the heart. The generation of these mediators is best investigated in the heart. In the ischemic heart, a mix of mediators including prostaglandins, prostacyclin, ATP, adenosine and particularly bradykinin is released in a compensatory attempt to improve perfusion [29,30,31,32,33,34]. Together with tissue acidosis, these mediators are supposed to cause anginal pain [33]. These vasoactive inflammatory mediators have algesic, hyperalgesic, spasmogenic and microvascular-leakage-inducing effects. PGE2 in particular may play a role in dysmenorrhea but it is probably only one of several mediators released. Its spillover into the systemic circulation causes effects remote from the uterus and is considered responsible for symptoms of fatigue, flu-like symptoms, fever (PGE2), edema and pain [25]. PGE2 also has awakening effects and sleep disturbances are present in dysmenorrhea. Probably due to the similarity of symptoms, many women with ME/CFS (more than one third) have a diagnosis of dysmenorrhea or endometriosis as well [6].

To summarize, in ME/CFS, inflammatory mediators are released from skeletal muscle, in endometriosis and dysmenorrhea from an ischemic uterus muscle, while in MCAS, mast cells are the source of vasoactive and inflammatory mediators. There is probably an overlap in the profiles of the mediators released in all three syndromes, which is probably most similar in ME/CFS and dysmenorrhea. Less overlap seems present between mediators in MCA and ME/CFS, where in the latter, bradykinin could be the predominant mediator, while it could be histamine in the former. Both key mediators, however, share common actions such as vasodilation, increase in vascular leakage, spasmogenic effects and some effects on sensory nerves. When these syndromes co-occur, the different types of mediators can act at the same time and often synergistically so that the clinical situation can result in an indistinguishable mixed or overlapping symptomatology. It is not surprising that orthostatic dysfunction is common among these syndromes as vasoactive mediators are released, perturbing vascular orthostatic regulation.

### 2.4. The Potential Role of Dysfunctional β2AdR

A further similar feature of the three syndromes could be the presence and importance of the beta-2 adrenoceptor (β2AdR) in the respective primarily affected organs or tissues. The importance of β2AdR dysfunction (autoantibodies, desensitization, polymorphisms) for the pathophysiology of ME/CFS is explained in our two first hypothesis papers [5,35]. β2AdR are important for the perfusion of skeletal muscles, the heart, and the brain by their vasodilator function. Cerebral and muscle malperfusion is a key element in our hypothesis not only for fatigue (mental and muscle fatigue) but for the presumed key disturbance in skeletal muscle. The second important function of β2AdR in skeletal muscle related to an energetic deficit in ME/CFS is the β2AdR agonistic stimulation of the Na^+^/K^+^-ATPase. Insufficient stimulation leads to an intracellular rise in sodium to a point where the sodium-calcium-exchanger (NCX) reverses its transport direction importing instead of exporting calcium with the consequence of functional damage by calcium overload [35]. This affects not only the mitochondrium but also the vascular endothelium in skeletal muscle perpetuating the disease via the production of ROS from the damaged mitochondrium. Hypoperfusion and disturbed metabolism together lead to an excessive metabolically driven generation of vasoactive tissue mediators with inflammatory properties for compensation resulting in a spillover into the systemic circulation so that any organ can be reached to produce the confusing myriad of symptoms.

β2AdR agonism is the most potent pharmacological uterus relaxing (tocolytic) mechanism. Dysfunctional β2AdR caused by autoantibodies against β2AdR [5,35,36,37,38,39,40] or desensitization by chronically elevated sympathetic activity (as present in ME/CFS) could cause stronger uterine contractions as contraction would not be mitigated by the relaxing effect of β2AdR agonism. This would raise intrauterine pressure to hamper uterine perfusion stimulating the production of ischemically released vasoactive mediators [5,35,39].

Mast cells are also endowed with β2AdR [41]. There is scarce literature on the importance of β2AdR agonism for mast cell degranulation, but epinephrine given in the intention to treat anaphylaxis is believed to impair mast cell degranulation via raising cAMP. Stress-induced mast cell degranulation is most likely mediated by the stimulation of alpha1-adrenergic receptors as alpha1-adrenergic receptor antagonism reduces mast cell degranulation, while blockade of the β2AdR enhances degranulation, suggesting that β2AdR stabilize mast cells [41]. Dysfunctional β2AdR could, therefore, play a detrimental role by favoring mast cell degranulation.

Hence, in all three syndromes, β2AdR dysfunction could be involved in the pathophysiology. We assume the strongest involvement in ME/CFS (condition sine qua non). In ME/CFS stress from different causes plays a role in initiation and perpetuation of disease. Once the disease is established, a high level of stress is generated and maintained by the disease state itself. Orthostatic stress could be the biggest stressor as it is already present in the sitting position in many patients suffering from ME/CFS [42].

### 2.5. Mutual Triggering and Disease Initiation

Post-exertional malaise (PEM) is an aggravation of the symptoms of ME/CFS and is already precipitated by minor everyday life or even mental efforts. Compared with such low levels of physical or mental efforts already causing PEM, the disturbance or stress imposed by a mast cell degranulation or dysmenorrhea could be far higher and big enough to also trigger symptoms. We assume that mast cell degranulation or severe dysmenorrhea can considerably worsen the symptomatology of ME/CFS. This not only has the potential to trigger aggravations or flairs of the disease but also to be one of several risk factors for the initiation of ME/CFS. Vice versa, ME/CFS has the potential to worsen mast cell degranulation and dysmenorrhea. As mentioned above, mast cell degranulation can also be triggered by stress [43,44]. Thus, the stress and sympathetic hyperactivity associated with ME/CFS could cause not only the desensitization of β2AdR in the uterus and on mast cells but also directly trigger degranulation via alpha1-adrenergic receptors [41]. Additionally, uterine contractility is also increased by alpha1-adrenergic receptor activation. The situation is reminiscent of the vascular situation in ME/CFS where vasoconstrictor influences are enhanced by sympathetic hyperactivity via alpha1-adrenergic receptors, but vasodilator effects are diminished by dysfunctional vascular β2AdR.

Concerning potential interactions between ME/CFS and dysmenorrhea, the association is not limited to β2AdR or to the generation of stress that could mutually affect both syndromes. For instance, bradykinin and PGE2 potentially released from skeletal muscle in ME/CFS could also favor uterine contractility and hyperalgesia [45] in other organs by spillover because of their known hyperalgesic and spasmogenic effects, including contractile effects on the uterus muscle. The hyperalgesic actions of such mediators can sensitize the pain receptors all over the body to the extent that otherwise subthreshold nociceptive stimuli then become painful. Since several mediators do have spasmogenic effects, even mild spasms in the presence of hyperalgesia may lead to overt pain in intestinal organs. Based on the idea of a mutual interaction, the question can be raised of whether dysmenorrhea is one of the factors that explains the higher prevalence of ME/CFS in women (an additional risk factor compared with men) [46,47].

**Figure 1:**
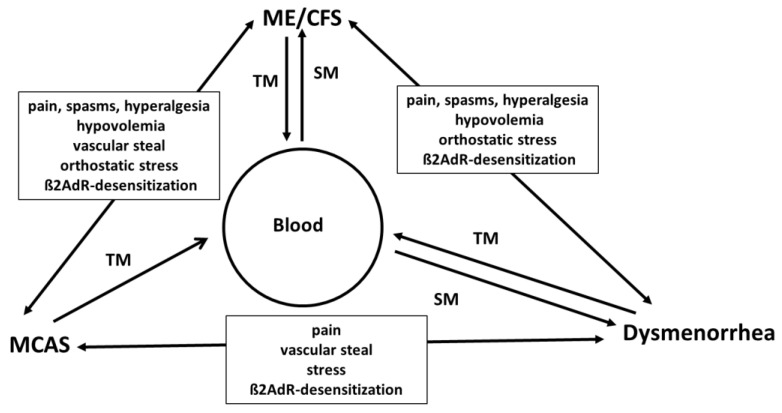
ME/CFS and associated diseases or syndromes. The three syndromes interfere with each other via multiple mechanisms. Pain is caused directly by the algesic effect of the mediators released into the blood stream indirectly through their hyperalgesic and spasmogenic effect. Negative hemodynamic effects include hypovolemia via microvascular leakage, renal excretory effects and negative effects on capacitance vessels causing orthostatic dysfunction and via intestinal hyperemia diverting blood from the brain and muscles. Pain and orthostatic dysfunction cause stress and desensitize **β2AdR** (beta2 adrenoceptor) relevant for the function of organs in the three syndromes. **TM:** tissue mediator, **SM:** mediator from spillover, orthostatic stress includes orthostatic intolerance (**OI**) and postural orthostatic tachycardia syndrome (**POTS**), **MCAS:** mast cell activation syndrome, and **ME/CFS:** Myalgic Encephalomyelitis/Chronic Fatigue Syndrome.

MCA and ME/CFS share many complaints and symptoms [43]. Due to the predominance of histamine and its sporadic release, MCA may be more anaphylactic or hypotensive, while in ME/CFS, preload failure is the dominant hemodynamic feature, presumably due to bradykinin and prostaglandins, and to a great extent due to their renal excretory actions. Based on the considerations made here we think MCA could create the conditions for ME/CFS and vice versa. Due to hypotension, hypovolemia, stress, and pain, misery perfusion of the skeletal muscles could develop to add other inflammatory vasodilators such as bradykinin released from the muscles to the inflammatory mediators already released by mast cells. Figure 1 shows the hypothesized pathogenesis of various syndromes associated with ME/CFS.

### 2.6. Disease Specific Symptoms versus Common Mechanisms

Symptoms specific for each syndrome most likely can be traced back to the respective affected tissue and tissue specific pattern of released mediators. In ME/CSF, where skeletal muscle is primarily affected, organ-specific symptoms are muscular pain, cramps, fatigue, weakness, and typically PEM. Examples for other disease-specific symptoms are pelvic (uterine) pain in dysmenorrhea and intestinal symptoms in MCA when histamine is released only by mast cells in the gut (as in the case of food intolerance) and when effects are limited to the affected organ, although ME/CFS patients may also independently present with some of these symptoms. However, mast cells are widely distributed, and degranulation, therefore, can take place in several tissues. Symptoms common to the three syndromes are most likely due to the spillover of tissue mediators into the systemic circulation exerting remote effects on organs not primarily affected. Since the spectrum of mediators released is very similar (e.g., prostaglandins) or overlapping or because different key mediators such as bradykinin and histamine share many physiological mechanisms, symptomatics can be very similar. This includes the induction of pain, hyperalgesia, edema, hypovolemia, and low-grade fever (action of PGE2). Headache, hyperalgesia, fatigue, brain fog, impaired cognition, and edema are reported in patients with these three diagnoses already in cases where there is no suspicion of any comorbidity.

## 3. Association with Postural Tachycardia Syndrome, Reduced Cerebral Blood Flow and Orthostatic Intolerance

Postural orthostatic tachycardia syndrome (POTS), orthostatic intolerance (OI), SFN and brain fog are often associated with ME/CFS, although these syndromes can also exist independently [48]. These syndromes are considered by us as risk factors, premorbid conditions or as part of the ME/CFS symptomatology.

Postural tachycardia syndrome (POTS) as a variant of orthostatic intolerance is common to both ME/CFS and MCA (Figure 1). Mast cell disorders are associated with decreased cerebral blood flow and SFN, typically also found in ME/CFS [49]. Since orthostatic dysfunction and a decrease in cerebral blood flow are related, we discuss them in a context. Orthostatic regulation is a highly regulated cardiovascular process. It is not surprising that it will be disturbed by the presence of an excess of vasoactive mediators such as bradykinin, histamine, prostaglandins, and prostacyclin in the systemic circulation. Vascular pathomechanisms related to orthostatic function include hypovolemia by microvascular leakage and renal hyperexcretion, insufficient contraction of capacitance vessels due to increased vasodilator action and the diversion of blood in single tissues (e.g., intestinal hyperemia). The importance of each pathomechanism may be different among the diseases. Autoimmunity could be a trigger for orthostatic dysregulation, and autoantibodies directed against several vasoregulative G-protein-coupled receptors are found in patients with ME/CFS [50] and long COVID [51,52,53]. Provided that these autoantibodies are functional, it is obvious to assume that vascular dysregulation will be the consequence.

Concerning the frequent association of POTS and ME/CFS, causality could be mutual [5]. Primarily present severe orthostatic dysfunction could favor ME/CFS by orthostatic stress as outlined above and, in turn, ME/CFS could aggravate orthostatic dysfunction by causing cardiac preload failure through microvascular leakage and the renal excretory effects of vasoactive mediators released from skeletal muscles in the process of functional sympatholysis and as compensatory mechanisms to the existing energetic deficit [5].

The association of MCA with POTS, as an example for orthostatic stress, may be via ME/CFS or separate. Histamine is probably the most important mediator released by overactivated mast cells. Upon a histamine discharge, the excess of histamine has the potential to worsen the orthostatic intolerance (OI) via several vascular mechanisms: (1) intestinal hyperemia and steal effect when dealing with intestinal releases, (2) a systemic vasodilating effect will disturb the highly coordinated orthostatic regulation, not only affecting arterial but even stronger venous vessels. The more potent vasodilating effect of histamine on veins as the most important capacitance vessels involved in orthostatic regulation has been explained by a higher histamine receptor density in veins compared to arteries and due to the fact that veins have a thinner smooth muscle layer than arteries, making them more responsive to histamine-induced relaxation [54,55,56,57,58,59,60], and (3) plasma exudation through microvascular leakage and the resulting hypovolemia.

Patients with ME/CFS show a reduction in absolute cerebral blood flow [61] and a decrease in cerebral blood flow during orthostatic challenge (head-up tilt testing), i.e., after leaving the horizontal position. In severe ME/CFS, this occurs already after changing the position from supine to sitting [62]. Concomitantly, disturbed neurovascular coupling and endothelial dysfunction are also present in ME/CFS [63,64,65]. In ME/CFS, hypovolemia and a predominance of vasoconstrictor over vasodilator influences by β2AdR dysfunction and other pathomechanisms of endothelial dysfunction cause cerebral vasoconstriction. Autonomic dysfunction could also be involved by overshooting responses of the sympathetic nervous system (vasoconstrictor overshoot) and of respiration (hyperventilation) [38]. The resulting hypocapnia enhances vasoconstrictor cerebral vascular tone.

OI in these syndromes is probably not transient after assuming the vertical position, but persists in the standing position, and in ME/CFS, even in the sitting position [42]. This causes permanent orthostatic stress which even has the potential to be a key factor in the initiation of the disease by desensitizing β2AdR and alpha2-adrenergic receptors, the two adrenergic receptors most sensitive to desensitization [66]. The desensitization of these inhibitory alpha2-adrenergic autoreceptors, otherwise limiting norepinephrine release in noradrenergic neurons in the CNS, could cause adrenergic overactivity, sympathetic hyperactivity, autonomic dysfunction and hypervigilance, explaining central symptoms such as hypersensitivities, difficulties to fall asleep and overshooting autonomic responses [38]. Hypervigilance increases neuronal energy demand but cerebral blood flow and, subsequently, energy supply are diminished, explaining the high level of fatigability at mental tasks, cognitive impairment and psychomotor slowing (brain overstimulated but hypoperfused, “tired but wired”). Brain fog according to our hypothesis is the consequence of overstimulation of the brain and hypoperfusion. The resulting energy deficit together with the non-specific overstimulation of the brain makes it impossible to concentrate on a single demanding mental task.

**Small fiber neuropathy (SFN)** associated with ME/CFS and MCAS could decrease the generation of neuropeptides such as Substance P and calcitonin-gene-related peptide (CGRP) released by these nerves. The ensuing deficiency of both vasodilator neuropeptides may contribute to skeletal muscle hypoperfusion. Moreover, CGRP is the second important stimulus of the Na^+^/K^+^-ATPase during exercise after β2-adrenergic stimulation. Insufficient stimulation of the ATPase by dysfunctional β2AdR and a lack of CGRP cause intracellular sodium accumulation in skeletal muscle and, subsequently, calcium overload. Calcium overload in turn causes mitochondrial and endothelial dysfunction [35]. The relationship of ME/CFS with SFN could be mutual. It could be a risk factor via the mechanisms just highlighted, favoring ME/CFS. The etiology of SFN is not fully understood. Again, autoimmunity, i.e., the presence of autoantibodies against these nerves, is a suspected pathomechanism among other mechanisms [67]. On the other hand, ME/CFS could cause SFN via a mechanism described in our first hypothesis paper [5]. Cerebrospinal fluid pressure is increased in ME/CFS [68,69]. An increase in blood–brain barrier permeability due to bradykinin could cause an excessive production in cerebrospinal fluid and from that a higher pressure, also in the Remak bundles of unmyelinated fibers (internal compression), potentially affecting perfusion, while bradykinin, histamine and PGE2 stimulating exactly this type of nerve fibers may simultaneously increase energy consumption so that a mismatch between energy consumption and blood supply appears, that could result in a chronic energetic deficit and chronic damage. In MCA, microvascular leakage may also damage small fiber nerves via similar mechanisms. How SFN could then act as a risk factor for ME/CFS has been explained above.

## 4. Conclusions

Our unifying hypothesis of the pathophysiology of ME/CFS provides clues on possible mechanisms linking ME/CSF with MCA, dysmenorrhea, POTS, decreased cerebral blood flow and small fiber neuropathy. We are convinced that in all these syndromes, similar pathomechanisms are operative that not only explain the causes of each disease but also their frequent association. These mechanisms include the excessive generation and spillover into the systemic circulation of inflammatory and vasoactive tissue mediators acting synergistically, dysfunctional β2AdR, and the mutual triggering of symptomatology and disease initiation. The comorbidity of ME/CFS and MCA seems particularly important in the pathophysiology of OI. The vast majority of ME/CFS suffers from OI and decreased cerebral blood flow. Comorbid mast cell degranulation has the potential to considerably worsen the pre-existing orthostatic dysfunction through the particular vascular effects of histamine. The detrimental vascular effects of histamine on orthostatic regulation and adequate cardiovascular adaptation to exercise include inadequate arterial vasodilation (steal effects), plasma exudation (hypovolemia), and, perhaps most important, the strong effect of histamine on veins as the main capacitance vessels (causing preload failure).

In all these associations, there is a strong involvement of vascular pathophysiology. Understanding the common mechanisms and differences between these syndromes also helps to better understand each of these syndromes.

## Data Availability

Not applicable.
